# Comparison of Heating Strategies on Soil Water Measurement Using Actively Heated Fiber Optics on Contrasting Textured Soils

**DOI:** 10.3390/s21030962

**Published:** 2021-02-01

**Authors:** Duminda N. Vidana Gamage, Hiteshkumar B. Vasava, Ian B. Strachan, Viacheslav I. Adamchuk, Asim Biswas

**Affiliations:** 1 Department of Natural Resources Sciences, McGill University, 21111 Lakeshore Road, Ste-Anne-de-Bellevue, QC H9X 3V9, Canada; duminda.vidanagamage@mail.mcgill.ca (D.N.V.G.); ian.strachan@mcgill.ca (I.B.S.); 2 Department of Soil Science, University of Peradeniya, Peradeniya 20400, Sri Lanka; 3 School of Environmental Sciences, University of Guelph, 50 Stone Road East, Guelph, ON N1G 2W1, Canada; hvasava@uoguelph.ca; 4 Department of Bioresource Engineering, McGill University, 21111 Lakeshore Road, Ste-Anne-de-Bellevue, QC H9X 3V9, Canada; viacheslav.adamchuk@mcgill.ca

**Keywords:** calibration, fiber optics, soil thermal properties, soil water, active heating, spatial variability

## Abstract

The actively heated fiber optics (AHFO) technique has the potential to measure soil water at high spatial and temporal resolutions, and thus it can bridge the measurement gap from point to large scales. However, the availability of power might restrict its use, since high power is required to heat long fiber optic cables under field conditions; this can be a challenge for long-term soil water monitoring under field conditions. This study investigated the performance of different heating strategies (power intensity and heating duration) on soil water measurement by the AHFO technique on three different textured soils. Different heating strategies: high power–short pulses (20 Wm^−1^–3 min), low power–short pulses (10 Wm^−1^–3 min, 5 Wm^−1^–3 min, 2.5 Wm^−1^–3 min) and low power–long pulses (10 Wm^−1^–5 min, 5 Wm^−1^–10 min, 2.5 Wm^−1^–15 min) were tested using laboratory soil columns. The study compared the sensitivity of the thermal response, NT_cum_ to volumetric water content (VWC) and the predictive error of different heating strategies and soils. Results of this study showed that the sensitivity of NT_cum_ increased and the predictive error decreased with increasing power intensity, irrespective of the soil type. Low power–short heat pulses such as 5 Wm^−1^–3 min and 2.5 Wm^−1^–3 min produced high predictive errors, RMSE of 5–6% and 6–7%, respectively. However, extending the heating duration was effective in reducing the error for both 10 and 5 Wm^−1^ power intensities, but not for the 2.5 Wm^−1^. The improvement was particularly noticeable in 5 Wm^−1^ –10 min; it reduced the RMSE by 1.5% (sand and clay loam) and 2.73% (sandy loam). Overall, the results of this study suggested that extending the heating duration of 10 and 5 Wm^−1^ power intensities can improve the sensitivity of the thermal response and predictive accuracy of the estimated soil water content (SWC). The results are particularly important for field applications of the AHFO technique, which can be limited by the availability of high power, which restricts the use of 20 Wm^−1^. For example, 5 Wm^−1^–10 min improved the predictive accuracy to 3–4%, which has the potential to be used for validating soil water estimations at satellite footprint scales. However, the effects of diurnal temperature variations should also be considered, particularly when using low power intensity such as 5 Wm^−1^ in surface soils under field conditions.

## 1. Introduction

Measurement, monitoring, and simulation of SWC and associated hydrological processes over a range of scales continue to be a great challenge due to soil water’s strong spatial and temporal variability, which results from the individual or combined effects of factors such as soil properties, topography, vegetation, climatic processes, groundwater, and water routing processes [1,2]. A range of point-based sensors (e.g., TDR, FDR, and capacitance probes) have advanced water measurement at the point scale, while remote sensing can estimate SWC at large scales (continental to global scales). Upscaling the point scale measurements to intermediate (e.g., field) and large scales requires substantial ground-based measurements. Several methods have emerged to measure SWC at the intermediate scales and bridge the gap between point to intermediate to large scales, e.g., cosmic ray probes [3,4], EMI sensors [5,6], global positioning system (GPS) reflectometry [7], and DTS [8,9,10,11].

A relatively new technique, DTS, measures temperature from the meter to kilometer scales with a very high temporal frequency (1 s), which has opened many possibilities for environmental monitoring. The DTS techniques used for measuring soil water can be broadly divided into two categories: AHFO and passive DTS. An electrically generated active heat pulse is generally sent through the optical fiber cable, which results in temperature change (thermal response) during (heating phase) or after (cooling phase) the heat pulse in the AHFO technique. The change in temperature is related to the amount of water in soil, and thus an empirically or physically based equation can be developed to quantify the relationships between temperature change and the soil water content [8]. However, on the other hand, passive DTS estimates soil water content using the soil’s thermal responses to the net solar radiation [9]. Studies have used the AHFO technique to measure soil water from the thermal conductivity (*λ*)–SWC relationship [12] or T_max_–SWC [13] relationship. However, with SWC estimation the errors were high (>0.05 m^3^m^−3^) for both methods in wet soils. Sayde et al. [8] introduced a new method called T_cum_ and found that SWC measurements are more precise (0.03 m^3^m^−3^) than the T_max_ and (*λ)* –SWC methods. Weiss et al. [11] and Perzlmaier et al. [12] used the long-time approximation of either the line-source or the cylindrical-source transient methods to calculate the thermal conductivity of the soil. It was concluded that (1) the method could only distinguish qualitatively between dry, wet, and saturated soils [11,12] and that (2) small changes in soil water content could not be detected at levels above 6% volumetric water content [11]. Weiss et al. [11] concluded that the substantial improvement in the signal to noise ratio of the DTS instrument is required to provide accurate thermal conductivity, which could be more sensitive to water content in soil. However, in this study, we used the T_cum_ as the thermal response, which is related to the signal magnitude of the DTS. T_cum_ is calculated by integrating the difference in temperature due to heating during a pulse, and the T_cum_ is the overall magnitude of the temperature change and was quite sensitive to moisture content [8]. Gil-Rodriguez et al. [13] also demonstrated that the T_cum_–SWC method provided satisfactory estimates of SWC distributions around drip emitters. The T_cum_–SWC method relates the direct DTS measurements (cumulative temperature increase) to independent SWC measurements, and it was found to be a highly sensitive function of SWC (i.e., the rate of heat exchange and the resulting integral also changes with SWC). Although the T_cum_–SWC method has reported better accuracy than the previous methods, studies by Sayde et al. and Gil-Rodriguez et al. [8,11] used high power intensity–short heat pulses (i.e., 20 Wm^−1^-2 min) in the laboratory. Further, extending the T_cum_–SWC method to observe the soil water variability along a 240 m long transect in the field, Sayde et al. [10] used heat pulses 10 Wm^−1^–1 min, and the results of the study demonstrated the need to use low-intensity pulses for longer cables in field applications.

Use of high power could be unrealistic in field applications, particularly when the available power supply is limited. T_cum_ is related to the signal magnitude of the measurement. The sensitivity of T_cum_ increases as the intensity of the heat pulse increases [8]. However, the sensitivity of T_cum_ can also increase due to the extended heating duration, because the DTS records the temperature based on the cumulative photon counting. The standard deviation of DTS temperature measurements reduces with the square root of the reading time [14]. Therefore, the T_cum_ obtained from a low power intensity–long heat pulse may have a similar sensitivity compared to that of high intensity–short heat pulse. Moderate power intensity–long heat pulses could be more feasible than the high-power heat pulses under limited power conditions in the field. Therefore, comparing the heating strategies is essential to find suitable heating strategies for field applications of the AHFO technique, particularly for long-term soil water monitoring. Recently, Li et al. [15] compared the performance of the T_cum_ method with other methods to measure SWC using a SPHP in different textured soils, but not using the AHFO technique. Dong et al. [16] compared T_cum_, T_max_, and *λ* methods using three heating strategies in sandy soil. However, studies that used the T_cum_ method widely varied in power intensity, heat pulse duration, and soil type, making the comparison difficult. It may be more convenient to compare the T_cum_ method for different heating strategies on contrasting textured soils using laboratory soil columns. A study by Cao et al. [17] used a carbon fiber heated sensing-tube (CFHST) integrated into conventional fiber optic sensing cable to improve the sensitivity, accuracy, and spatial resolution of the measurement of soil moisture profile. It was concluded that the new method (CFHST) measured the soil moisture profile accurately (RMSE = 0.05 m^3^m^−3^), and the method can capture the continuous soil moisture profile along the depth direction. Further, Kurashima et al. [18] introduced a new approach to AHFO technique by integrating Brillouin optical time domain analysis (BOTDA) technology for monitoring soil moisture distribution as well as strain distribution. In this study, we developed a three-dimensional measurement network by wrapping the fiber optic cable into two helical coils in repacked soil columns. The three-dimensional measurement network allowed the measurement of SWC across a wider range to develop calibration relationships. The main objective of this study was to investigate the performance of different heating strategies (power intensity and heating duration) on soil water measurement using the AHFO technique on three different textured soils. It was assumed that low power intensity–long heat pulses could also produce similar accuracies of the measured water content like the high-power intensity–short heat pulses, regardless of the soil type. The results would be particularly important if the AHFO technique were to be a tool for measuring SWC at field scale over a range of soils.

## 2. Materials and Methods

### 2.1. Soil Column Construction

**Figure 1:**
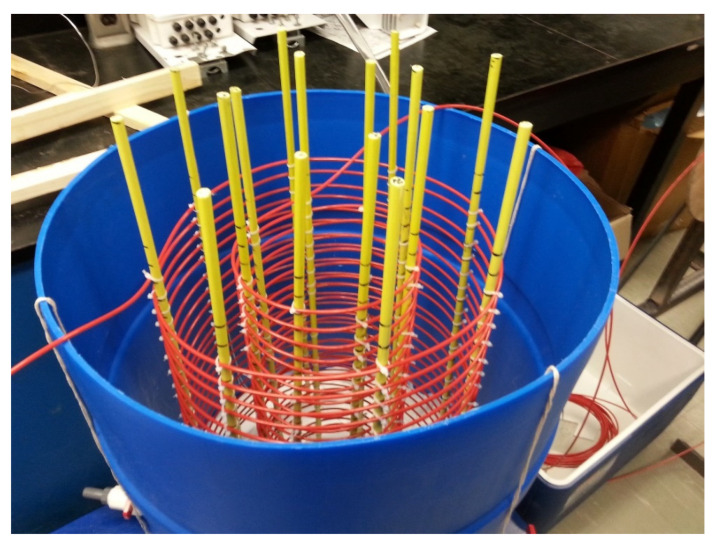
The column structure developed using a plastic barrel, inner and outer cable helices, and fiber glass rods supporting the cable helices.

Three soils, sand (mean diameter 0.26 mm), sandy loam, and clay loam (Table 1), were used in this experiment to develop soil column. A plastic barrel (height of 0.85 m and a diameter of 0.6 m) was used to develop an artificial soil column and was used for all individual soils. To create an open or free-flow bottom boundary condition, a plexiglass base with small perforations (0.005 m in diameter) was set at a height of 0.15 m from the bottom of the column. Two layers of gravel and stones to a maximum height of 0.2 m in total were placed on the Plexiglas base to facilitate free drainage from the column. A water release tap was attached at the bottom of the coil column. A long (length 44.5 m) fiber optic cable was used to develop a soil water measurement network inside the barrel. Two concentric helixes with diameters of 0.10 and 0.20 m (Figure 1) and spacing of 0.025 m between the turns were developed to form a 3D network of 178 measurement points along the fiber optic cable. A set of ten fiber glass rods (0.005 m diameter) were attached to the bottom plexiglass base to support the helixes. The barrel was filled with respective soil at corresponding bulk densities (Table 1). A total weight of 238.80, 217.10, and 202.60 kg of sand, sandy loam, and clay loam soils, respectively, were added into the barrel to pack soil column. The required soil amount was calculated based on the volume of the barrel minus the volume of fiber glass rods and the set bulk density. To reduce nonuniform packing, an amount of about 20 kg (~0.05 m height) soil was added to the column using a controlled lift.

### 2.2. Temperature Measurement

**Figure 2:**
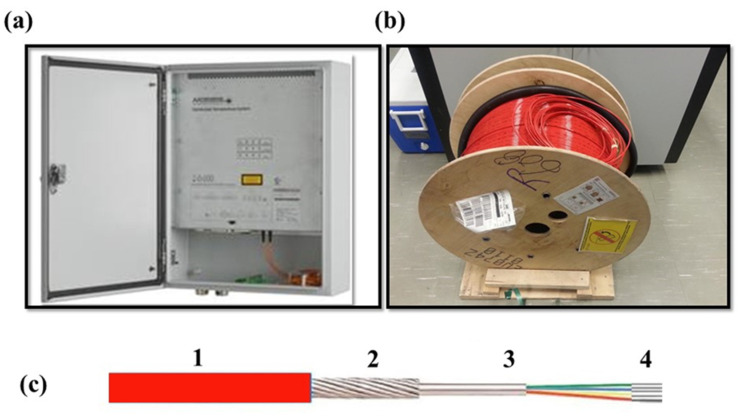
(**a**) The DTS instrument and the (**b**) fiber optic cable spool used in this experiment. The BRUsteel fiber optic cable composition is shown in (**c**), including (1) the nylon jacket, (2) the inter-laced steel wires, (3) the stainless-steel loose tube filed with gel, and (4) the four multimode fibers. The information collected from the technical spec-sheet of the BRUsteel fiber cable (http://www.bruggcables.com).

A DTS (model: Linear Pro series) from AP Sensing, Germany, was used in this experiment. The DTS model has two channels, and the maximum measurement range is 4 km with 0.5 m spatial resolution of recording (integrated length of measurement of a single value of temperature) and 0.25 m spatial resolution of sampling (the minimum distance between two consecutive measurement points) (Figure 2a). A special model of fiber optic cable was purchased from BRUsteel, Brugg Cable, 120 Switzerland (Figure 2b). The cable consists of four multimode 50 μm cores and 125 μm cladding fibers within a stainless-steel loose tube. This tube is further surrounded by stainless steel strands and a protective nylon jacket (Figure 2c). All these four components of the cable make the external cable diameter of 3.8 mm. The DTS instrument generates a laser pulse, which generally travels along the fiber optic cable. During the travel, the photons and electrons collide with each other and result in backscattered Raman Stokes and anti-Stokes photons in the core of the glass fiber. The ratio of Stokes to anti-Stokes and the elapsed time between the emitted laser light and the returned light are then used to estimate the temperature. For more details on the principle of temperature measurement using DTS, please refer to Kurashima et al. [17] and Tyler et al. [19], and for its application for environmental temperature monitoring, please refer to Selker et al. 2006a and 2006b [14,20].

**Figure 3:**
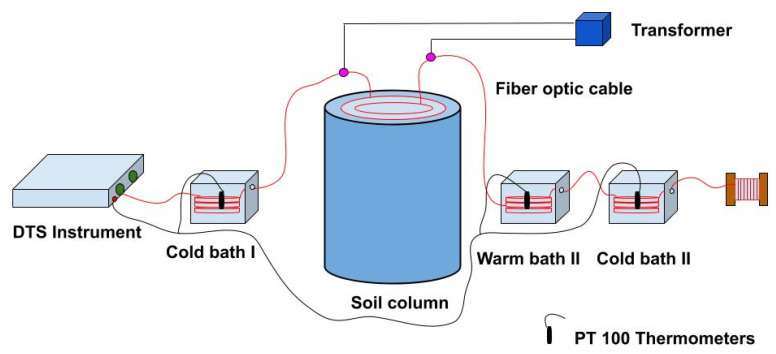
Diagrammatic view of the experimental set up. It shows the single-ended cable configuration with three temperature calibration baths with PT 100 thermometers, DTS instrument, soil column, and other components. Electrical connections were made to the fiber optic cable to send pulse, and the purple circles show the connection locations. The locations of electrical connections on the fiber optic cable were just before entering the cable and after the coming out of the cable from the soil column.

An independent calibration set up was developed using three sections of unburied fiber optic cable to calibrate the DTS recorded temperature. The unburied cables were coiled in two cold and one warm bath (Figure 3), and platinum resistance thermometers (PT100, AP Sensing, Böblingen, Germany) were used to measure temperatures of the calibration baths. Aquarium pumps were used to circulate water within the calibration baths to ensure the uniformity of the temperature within the calibration bath. A single ended measurement protocol was adopted in this study, where laser pulses were sent through one end of the cable attached to the DTA. For DTS temperature calibration, we followed the procedure described by Hausner et al. [21]. Based on the locally measured Stokes and anti-Stokes signals, temperature at each sampling point was calculated as

(1) $$T\left(z\right)=\frac{\;\gamma }{\ln\frac{P_{s}\left(z\right)}{P_{\mathrm{as}}\left(z\right)}+C-\Delta \alpha z}$$

where γ is the shift in energy between two photons, incident laser and the scattered Raman photon; C is the time dependent calibration parameter; and Δα represents the differential attenuation between the anti-Stokes and Stokes signals in the fiber.

### 2.3. Heat Pulse Experiment

**Figure 4:**
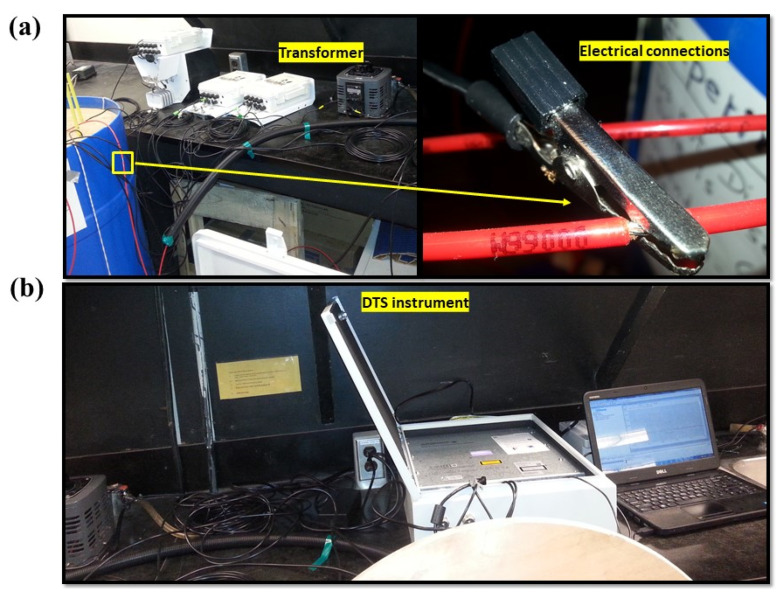
(**a**) An electrical connection (from one side) used to heat up the fiber optic cable, and the variable transformer, which was used to generate the electricity required for different power levels, (**b**) the DTS instrument connected to the computer to record the temperature during the experiment.

To set up the heat pulse experiment, an electrical connection was made to the fiber optic cable. Two small battery clamps were connected to the metal sheath of the fiber optic cable after removing a few centimeters of the protective nylon jacket. The connections were made at two locations on the cable immediately before it enters into the soil and after coming out of the soil (Figure 4a). The applied heat pulse of different power intensities was controlled using a variable transformer (TDGC-0.5KVA, Variac, Cleveland, OH, USA). A power control unit was developed using a microcontroller (ATmega328P, Arduino Uno, Somerville, MA, USA) and a relay board, and the heat pulse duration was controlled to an accuracy of 0.001 s. Seven heating strategies (seven pairs of power intensity–hating duration) were tested (Table 2). The DTS instrument measured the temperature of every 0.25 m length of the fiber optic cable at 30-s intervals, and they were recorded on a computer (Figure 4b). After filling the soil column, it was allowed to remain unaltered for approximately two weeks to observe any possible subsidence. Then, a water reservoir below the Plexiglas base of the column (bottom) gradually wetted the soil column. The wetted soil column was then gravity drained over almost a two-month period. The gradual and natural drain of soil water helped establish a soil water gradient in the column. A three-step sampling procedure was used to measure SWC for the calibration and validation experiment. Before each sample step, all seven pairs of power intensity–heating duration were sent at 20 min intervals. For example, the first heat pulse applied used a power intensity of 2.5 Wm^−1^ with a heating duration of 3 min. Then, the power intensity was doubled, and the same heating duration (3 min) was used. This was continued at 10 and 20 Wm^−1^ with the same heating duration of 3 min; 2.5 and 20 Wm^−1^ were the lowest and highest power intensities used. In the second round, the heating duration of each power intensity was increased except for 20 Wm^−1^ (Table 2). Immediately after the heat pulse experiment, 33 volumetric soil samples were collected from 0–0.20 m depth. After removal of the disturbed soil of 0–0.20 m, the remainder of the soil column was allowed to dry for one week drying, and the heat pulse experiment was repeated before the second sampling and third sampling steps. Thirty-three samples from 0.2–0.4 m and 18 samples from 0.4–0.5 m depths were taken in the second and third steps, respectively. The sampling procedure was very helpful to obtain SWC data across a wider range, especially for clay loam soil, which took a relatively longer time period to drain naturally. A set of 84 volumetric soil samples were collected from the soil column at the end of the experiment. After every sampling step, soil samples were immediately transferred to an oven, and SWC of each sample was determined following gravimetric measurement technique after drying them in a hot air oven at 105 °C for 24 h. This procedure obtained SWC measurements across a wide range required for calibration and validation. During a heat pulse experiment, SWC within the soil column remained unchanged as monitored by five commercial soil water measurement sensors (SMEC 300, Spectrum Technologies, Inc., Plainfield, IL, USA). These SMEC300 sensors were installed at 0.1, 0.2, 0.3, 0.4, and 0.5 m depths, separated by approximate distance of 0.035 and 0.06 m from the inner and outer helices, respectively.

### 2.4. Data Analysis

The integral of the cumulative temperature increase (T_cum_) during a heat pulse [8] was calculated at each point of the fiber optic cable using

(2) $$T_{\mathrm{cum}}=\overset{t_{0}}{\underset{0}{\int }}\Delta \mathrm{Tdt}$$

(3) $${\mathrm{NT}}_{\mathrm{cum}}=\frac{{\mathrm{NT}}_{\mathrm{cum}}}{q}$$

where T_cum_ is the cumulative temperature increase (˚C s) over the whole measurement time t_0_ (s) at a given point of the cable. ΔT is the change in temperature measured using DTS from the pre-pulse temperature (˚C). T_cum_ is a function of soil thermal properties including thermal conductivity. Generally, higher thermal conductivity from high SWC will lead to a low T_cum_ at a given point on the cable, as the heat will be carried away from the cable. The pre-pulse temperature in this study was calculated as the average temperature over five minutes before the start of the heat pulse. This average temperature was then subtracted from the measured temperature during the pulse to estimate the increase in temperature ΔT. The obtained ΔT values were then multiplied by the time interval (30 s) between measurements to calculate T_cum_ in this study. Following this, T_cum_ was calculated for every heat pulse at each point of the fiber optic cable within the soil column. The power intensity (q) corresponding to each heating strategy was used to convert T_cum_ to NT_cum_ using Equation (3).

A total of 84 SWC measurements obtained from the gravimetric method were halved: 42 calibration SWC measurements and 42 validation SWC measurements. It was ensured to include SWC data obtained from the three sampling steps explained in Section 2.3 to both calibration and validation data sets. This procedure allowed the inclusion of SWC data across a wider range (from wet to dry) into both calibration and validation data sets. Forty-two calibration SWC measurements and corresponding NT_cum_ measurements from a given heating strategy were used to develop a calibration curve of that heating strategy. Accordingly, seven calibration curves were developed for a given soil. SWC was subsequently predicted using calibration curves, and predictions were validated using the 42 validation SWC measurements. The root mean squared error (RMSE) was used to evaluate (N = 42) predictive accuracy of the calibration relationships following Equation (4).

(4) $$\mathrm{RMSE}=\;\frac{1}{n}\sqrt{\overset{n}{\underset{i=1}{\sum }}{\left(T_{i}-T_{\mathrm{oi}}\right)}^{2}}$$

where T_i_ and T_oi_ are the AHFO technique estimated and gravimetric method measured SWC, respectively, and n is the number of observations (42).

## 3. Results

### 3.1. Comparison of T_cum_–SWC Relationships

**Figure 5:**
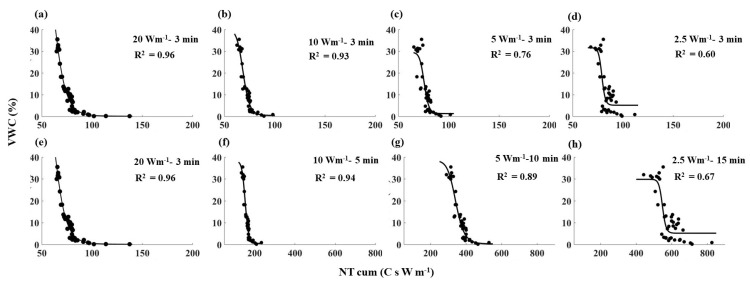
NT_cum_ as a function of VWC for all the heating strategies tested in sandy soil. The black dots show the observed values, and the black solid lines show the fitted relationships between NT_cum_ and VWC for individual heating strategies. Note: (**a**,**e**) are similar (20 Wm^−1^–3 min) and used to compare with the short pulses (along the row 1) and with the long pulses (along the row 2).

**Figure 6:**
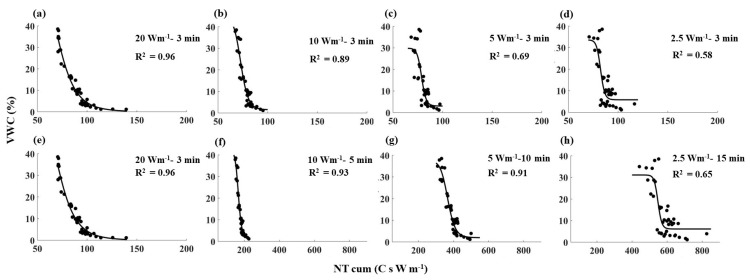
NT_cum_ as a function of VWC for all the heating strategies tested in sandy loam soil. The black dots show the observed values, and the black solid lines show the fitted relationships between NT_cum_ and VWC for individual heating strategies. Note: (**a**,**e**) are similar (20 Wm^−1^–3 min) and are used to compare with the short pulses (along row 1) and with the long pulses (along row 2).

**Figure 7:**
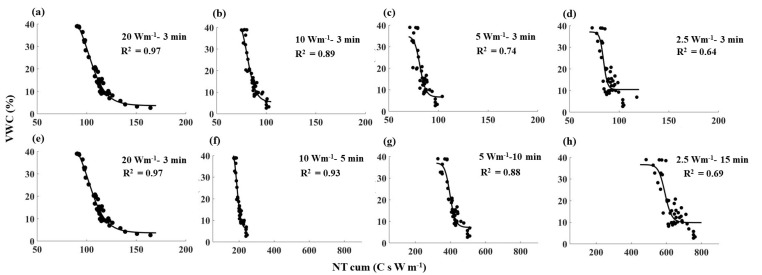
NT_cum_ as a function of VWC for all the heating strategies tested in clay loam soil. The black dots show the observed values, and the black solid lines show the fitted relationships between NT_cum_ and VWC for individual heating strategies. Note: (**a**,**e**) are similar (20 Wm^−1^–3 min) and are used to compare with the short pulses (along row 1) and with the long pulses (along row 2).

Despite the differences in absolute values, all the NT_cum_–SWC relationships showed a similar shape; NT_cum_ decreased with increasing SWC, and the rate of decrease was relatively low when the SWC was below 5% (Figure 5, Figure 6 and Figure 7). The decrease in NT_cum_ was due to elevated heat transfer in the soil as SWC % increased, resulting in a small temperature rise in the cable. When the soil is dry (e.g., <5%), it was not sufficient to bridge the air gaps between soil particles. Therefore, the heat transfer was less rapid, causing NT_cum_ to decrease at a slower rate. When the soil became progressively wet, elevated heat transfer resulted in a rapid decrease in NT_cum_.

The sensitivity of NT_cum_ to SWC followed the order of 20 > 10 > 5 > 2.5 Wm^−1^ for 3 min heating duration in any soil type. For example, high power intensity–short pulse (20 Wm^−1^–3 min) had the highest R^2^ values in all three soils (Figure 5a, Figure 6a and Figure 7a), which indicated the high sensitivity of T_cum_ to SWC. For a given soil, the magnitude of NT_cum_ was highest for 20 Wm^−1^–3 min; it decreased to around 100 C s W m^−1^ when power intensity decreased with the same heating duration of 3 min. NT_cum_ related to the signal magnitude, and thus it increased with increasing temperature of the fiber due to the high-power intensity used. The sensitivity of NT_cum_ to SWC was poor when low power and short pulse (e.g., 2.5 Wm^−1^–3 min) was used for any soil type. Maximum R^2^ reported was 0.64 for clay loam soil (Figure 7), which suggests that the NT_cum_ was a weak function of SWC when low power and a short pulse were used. The sensitivity of NT_cum_ was also higher for 10 Wm^−1^–3 min and marginally higher for 5 Wm^−1^–3 min (Figure 5, Figure 6 and Figure 7).

Extending the heating duration increased the sensitivity of NT_cum_ substantially for both 10 and 5 W m^−1^ power intensities for the three soils, and the increment was relatively higher in 5 Wm^−1^ (Figure 5g, Figure 6g and Figure 7g). The increase in sensitivity could be attributed to the relatively longer heating duration of (10 min) in 5 Wm^−1^ compared to 5 min heating duration in 10 Wm^−1^. However, the sensitivity of NT_cum_ of 2.5 Wm^−1^ only marginally increased with increasing heating duration from 3 min to 15 min. The NT_cum_ was a weak function for all the three soils when 2.5 Wm^−1^ power intensity was used, irrespective of any increase in heating duration.

### 3.2. Comparison of Predicted Soil Water Content

**Figure 8:**
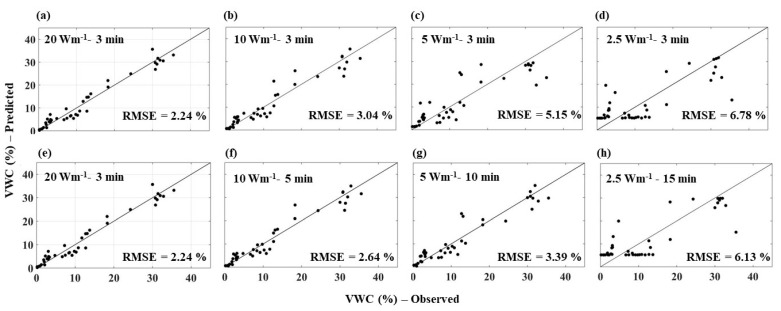
Comparison between AHFO predicted and observed (gravimetric method) soil water content of sandy soil for all the heating strategies. Note: (**a**,**e**) are similar (20 Wm^−1^–3 min) and are used to compare with the short pulses (along row 1) and with the long pulses (along row 2).

**Figure 9:**
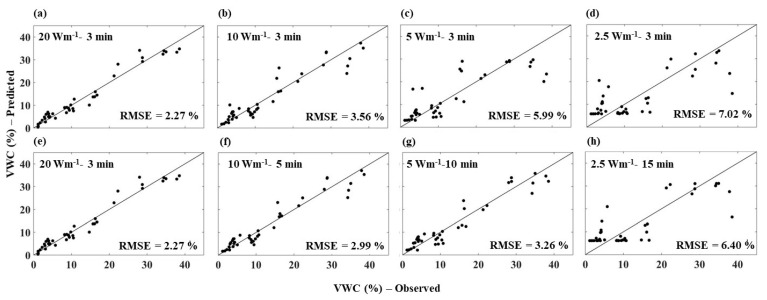
Comparison between AHFO predicted and observed (gravimetric method) soil water content of sandy loam soil for all the heating strategies. Note: (**a**,**e**) are similar (20 Wm^−1^–3 min) and are used to compare with the short pulses (along row 1) and with the long pulses (along row 2).

**Figure 10:**
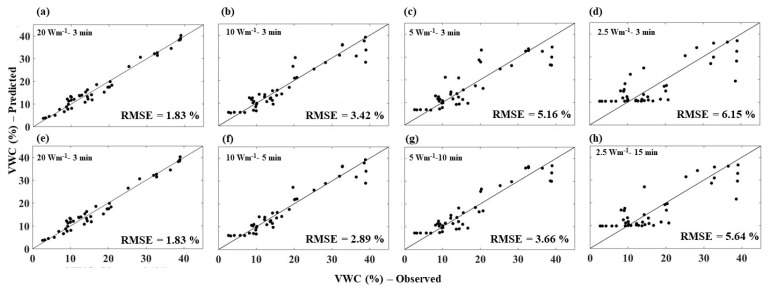
Comparison between AHFO predicted and observed (gravimetric method) soil water content of clay loam soil for all the heating strategies. Note: (**a**,**e**) are similar (20 Wm^−1^–3 min) and are used to compare with the short pulses (along row 1) and with the long pulses (along row 2).

In this section, the AHFO technique predicted SWC data were compared with corresponding measured SWC data by the gravimetric method for individual heating strategies. RMSE values indicated the predictive error of the AHFO technique compared to the gravimetric method. A high RMSE value indicated a larger error in predictions compared to the gravimetric method. Additionally, the spread around the 1:1 line indicted the agreement between the AHFO technique predicted and gravimetric method measured SWC. The RMSE increased as the power intensity decreased, but with the same heating duration (i.e., 3 min) for all soils. The spread around the 1:1 line indicated a relatively larger error in wetter soils (Figure 8, Figure 9 and Figure 10), which was comparable with previous studies [8,12,13]. The larger error was more noticeable for low power intensity–short pulse strategies of 5 and 2.5 Wm^−1^–3 min. Extending the heating duration was effective in reducing the error for both 10 and 5 Wm^−1^ power intensities. For example, the magnitude of the RMSE was reduced by 0.53% (sand), 0.55% (sandy loam), and 0.40% (clay loam) and 1.5 (sand and clay loam) and 2.73% (sandy loam), respectively, for 10 and 5 Wm^−1^. However, both 2.5 and 2.5 Wm^−1^–15 min had the highest predictive error. 

## 4. Discussion

Results of the study clearly showed that the behavior of thermal response (NT_cum_) was more or less similar irrespective of the soil type for a given heating strategy. The sensitivity of NT_cum_ associated with the magnitude of the signal, which increases with the fiber temperature. Therefore, highest power intensity, 20 W m^−1^, showed strongest relationship between NT_cum_ and SWC, while lowest power intensity (2.5 W m^−1^) showed weakest relationship between NT_cum_ and SWC for any given soil type. A study by Dong et al. [16] found that increasing heat pulse duration can produce similar improvements in sensitivity as an increase in power. However, the results of our study indicated that increasing the heating duration only reduced the error to a certain threshold of power intensity level; beyond this level, extending the heating time showed no effect. The low power intensity was inadequate to provide a substantial change in temperature compared to pre-pulse temperature. Therefore, the pre-pulse temperature error led to masking the SWC information contained in the heat pulse of 2.5 Wm^−1^. As the water content in soil increased (near saturation), an increase in the heat transfer in soil is less rapid, resulting in the NT_cum_ being less sensitive to the actual changes in SWC. Therefore, relatively larger errors in wetter soils irrespective of the heating strategy and soil type were observed. Interestingly, for the 2.5 Wm^−1^–3 min and 2.5 Wm^−1^–15 min, the error continued to be higher across the whole SWC range (scattered data points) (Figure 8d,h, Figure 9d,h and Figure 10d,h), which indicated the mix effect of the poor sensitivity and soil wetness. Results of this study could be useful particularly for field applications of the AHFO technique. For example, results of this study demonstrated that increasing the heating duration of moderate power intensity (e.g., 5 Wm^−1^) could significantly reduce the predictive error (3–4%), which is suitable for validating SWC estimations from remote sensing in contrasting textured soils. Nevertheless, use of medium power intensity level of 10 Wm^−1^ with increasing heating time is also possible on relatively shorter fiber optic cables (<1000 m) at the plot to field scales. The AHFO technique has the potential to examine the finer scale spatial structure of soil water at the field scale. However, it should be noted that this experiment was conducted in the laboratory, under the controlled conditions. Effects of diurnal temperature variations should also be considered, particularly when using the low power intensity such as 5 Wm^−1^ under field conditions. Background temperature correction approaches could improve the accuracy of SWC estimations under such conditions [16].

## 5. Conclusions

This study examined the performance of seven heating strategies (power intensity–heating duration) to measure SWC in three contrasting soils using the AHFO technique. The sensitivity of the thermal response, NT_cum_, and the predictive error (RMSE) were compared among the heating strategies and soils. Results of this study showed that the sensitivity of NT_cum_ increased and the predictive error decreased with increasing power intensity, irrespective of the soil type. Further, results of this study showed that extending the heating duration of moderate power intensities (e.g., 5 and 10 Wm^−1^) reduced the predictive error of the measurement, while the improvement was marginal for the lowest power intensity (2.5 Wm^−1^) irrespective of the soil type. Overall, the results suggested that extending the heating duration to improve the measurement accuracy was only feasible up to a threshold power intensity (e.g., 5 Wm^−1^). Findings of this study are important to design the power supply protocols to achieve a reasonable compromise between accuracy and practicality of soil water measurement using the AHFO under field conditions.

## Figures and Tables

**Table 1:** Sand, silt, clay percentages, and bulk densities of three soils used in the study.

Soil Type	Sand (%)	Silt (%)	Clay (%)	Bulk Density (Mg m^−3^)	Total Organic Carbon (%)
Sand	100	-	-	1.65	-
Sandy loam	67	21	12	1.50	2.65
Clay loam	45	19	36	1.40	3.24

**Table 2:** Seven heating strategies and their power intensities and heating durations.

Heating Strategy	1	2	3	4	5	6	7
Power intensity (W m^−1^)	2.5	5	10	20	2.5	5	10
Heating duration (min)	3	3	3	3	15	10	5

## Data Availability

Data can be available on request to authors.
